# Cytological study on the regulation of lymphocyte homing in the chicken spleen during LPS stimulation

**DOI:** 10.18632/oncotarget.14502

**Published:** 2017-01-04

**Authors:** Qian Zhang, Yasir Waqas, Ping Yang, Xuejing Sun, Yi Liu, Nisar Ahmed, Bing Chen, Quanfu Li, Lisi Hu, Yufei Huang, Hong Chen, Bing Hu, Qiusheng Chen

**Affiliations:** ^1^ Laboratory of Animal Cell Biology and Embryology, College of Veterinary Medicine, Nanjing Agricultural University, Nanjing, China; ^2^ Key Laboratory of Antibody Techniques of Ministry of Health, Nanjing Medical University, Nanjing, China; ^3^ Biological experiment and Teaching Center, College of Life Sciences, Nanjing Agricultural University, Nanjing, China

**Keywords:** lymphocyte homing, chicken spleen, HEV, sheathed capillary, adhesion molecules, Immunology and Microbiology Section, Immune response, Immunity

## Abstract

The immune function of the chicken spleen depends on its different compartments of red and white pulps, but little is known about the mechanism underlying lymphocyte homing towards the different compartments. In the present study, the role of lymphocyte homing in the chicken spleen was investigated during lipopolysaccharide (LPS) stimulation. Morphological analysis demonstrated the cuboidal endothelial cells of the splenic sheathed capillary facilitated the passage of lymphocyte homing to the chicken spleen. The tissue-specific adhesion molecules- vascular cell adhesion molecule-1 (VCAM-1) and mucosal addressin cell adhesion molecule-1 (MADCAM-1) expressed on the sheathed capillary, which suggested the high endothelial venule (HEV)-like vessels of the chicken spleen. Electron microscope analysis showed LPS activated the endothelium of the sheathed capillary and recruited lymphocytes to the chicken spleen. Transferring of 5, 6- carboxyfluorescein diacetate, succinimidyl ester (CFSE) labeled lymphocytes depicted the rout of lymphocyte homing to the compartments of the chicken spleen was from the white pulp to the red pulp. Furthermore, the mRNA and protein levels of adhesion molecular integrin β1 and VCAM-1 increased after LPS stimulation. The mechanism underlying the integrin β1 and VCAM-1 during LPS stimulation might be associated with the integrin linked kinase (ILK)- dependent regulation of protein kinase B (PKB/AKT). This study firstly shows lymphocyte homing in the chicken spleen after LPS-induced inflammation. These results contribute to our knowledge of comparative immunology and provide a better means for investigating the pharmacological strategies concerning the possible role of lymphocyte homing in inflammation and immunological reactions in infectious disease.

## INTRODUCTION

As the largest peripheral lymphoid organ, the spleen plays a significant role in antibacterial and antiviral immune reactivity. The chicken immune system has a crucial position in phylogeny as it is without lymph nodes but does have the bursa of Fabricius [1]. The structural composition of immune organs varies between avians and mammals. The mammalian spleen consists of red and white pulps with a marginal zone of separation [2, 3]. However, in chickens, there is no morphologically distinguished marginal zone [4, 5]. The white pulp in the chicken spleen includes three lymphoid regions: (1) the periarteriolar lymphocyte sheaths (PALS), which is the lymphoid tissue of the T lymphocytes that surrounds the central arteries; (2) the periellipsoid lymphocyte sheaths (PELS), which is the lymphoid tissue of the B lymphocytes that surround the branching penicillary capillaries; and (3) the lymph nodules which is the site of B lymphocytes proliferating and differentiating. The white pulp in chickens plays a crucial role in the initiate immune response especially against blood-borne antigens [6, 7].

Lymphocytes from the blood regularly migrate to the peripheral lymphoid organs and back to the blood every day. The continuous recirculation of lymphocytes into these immune organs is essential for immune surveillance. Lymphocytes migrate into the peripheral lymphoid organs, such as the lymph node and the Peyer's patch, *via* the specialized high endothelial venule (HEV) [8–10]. In mammals, the spleen lacks HEVs, which is the pathway for lymphocytes to enter to the spleen *via* the marginal zone and is an important area for lymphocytes that leave the bloodstream and enter the white pulp [11–13]. However, the structural organization of the chicken spleen is devoid of the marginal zone. How do lymphocytes migrate to the chicken spleen? Previous research identified the blood-spleen barrier of chickens, which is located in the antigen-trapping zone of the PELS and the ellipsoid [14], and a morphological study suggested that the sheathed capillary was a high endothelial venule (HEV)-like vessel. Whether the HEV-like vessels are implicated in lymphocyte homing to the chicken spleen is unknown.

Lipopolysaccharide (LPS) induces an immune response in normal animal immune systems and is an acceptable administration for studying systemic inflammation [15, 16]. The LPS-induced inflammatory immune response is mediated through the Toll-like receptor pathway, resulting in the increased expression of cytokines, such as IL-6 and TNF-α [17]. In mammals, LPS regulated the distribution of the marginal zone B lymphocytes in the spleen and increased the adhesion and migration of lymphocytes in blood-brain barrier [2, 18]. LPS stimulation of leukocytes also activates integrins β_1_ and β_2_ [19]. However, the adhesion molecules of the lymphocytes recognized the vascular addressins on the endothelium are tissue-specific [20–24]. For example, integrins LFA-1 (αLβ2), α4β7 and VLA-4 (α4β1) participate in lymphocyte homing to the peripheral lymph node and the gut-associated lymphoid tissue, but integrin α4β7 is not involved in lymphocyte homing to the bronchus-associated lymphoid tissue [25–29]. The ligands for LFA-1 are ICAM-1, ICAM-2 and ICAM-3; whereas, the VLA-4 (α4β1) and α4β7 ligands are vascular cell adhesion molecule-1 (VCAM-1) and mucosal addressin cell adhesion molecule-1 (MADCAM-1) [11, 30, 31]. The mechanism underlying the inflammatory-induced lymphocyte homing to the chicken spleen remains unknown. To further understand the regulated adhesion molecules involved in lymphocyte homing to the chicken spleen, we investigated the morphological basis, the migration pattern and the gene and protein expression of lymphocyte homing-related adhesion molecules in the chicken spleen. These data contribute to a better understanding of comparative immunology and therapy for abnormal lymphocyte homing caused by infectious diseases.

## RESULTS

### Morphological structure of the sheathed capillary in the chicken spleen

**Figure 1:**
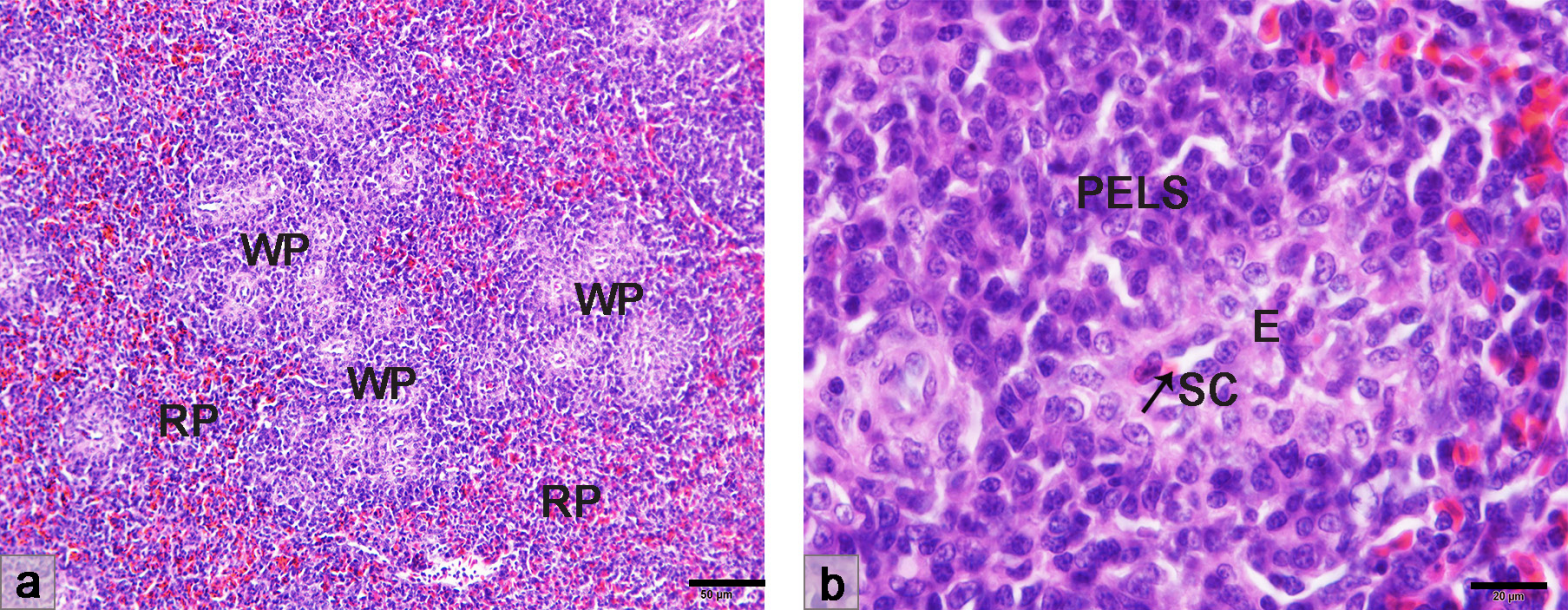
Histological structure of the chicken spleen with HE staining **a**. The compartments of the chicken spleen are red pulp and white pulp. **b**. The ellipsoid structure surrounding the sheathed capillary is encircled by the PELS. WP, white pulp; RP, red pulp; PELS, periellipsoidal lymphatic sheath; SC, sheathed capillary; E, ellipsoid.

The chicken spleen was histologically divided into the white pulp and the red pulp (Figure 1a). No identifiable marginal zone was observed in the chicken spleen. The PELS of chicken white pulp was the lymphoid tissue which surrounded the ellipsoid structure. The part of the penicilliform capillary surrounded by the ellipsoid was known as the sheathed capillary (Figure 1b).

**Figure 2:**
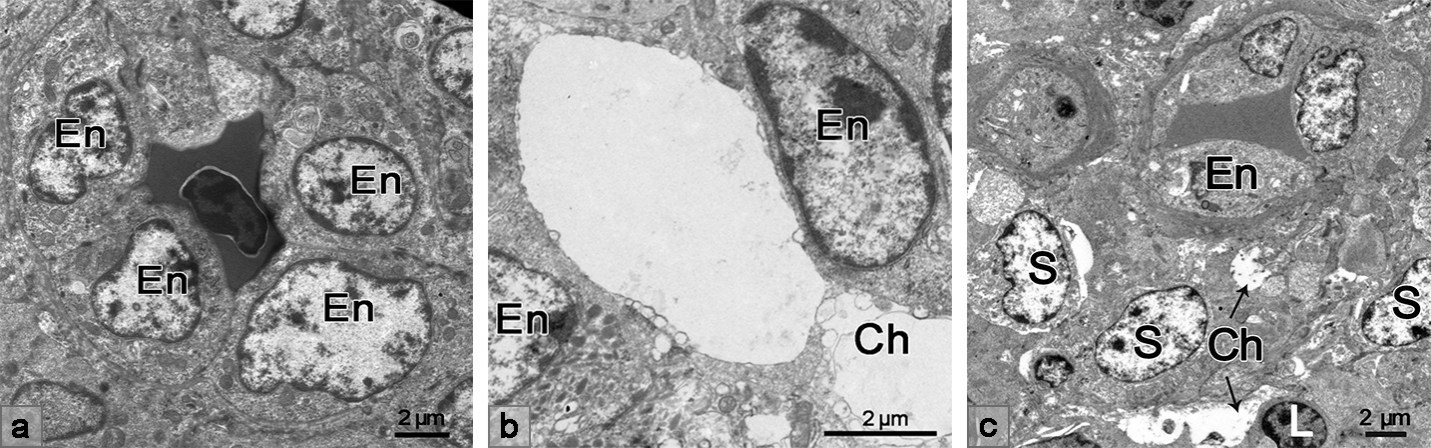
TEM showing the morphological structure of the sheathed capillary **a**. Endothelial cells of the sheathed capillary were plump and cuboidal in appearance. **b**. The vascular channel presented between the adjacent endothelial cells **c**. The lymphocyte appeared in the spleen ellipsoid along with the vascular channel. En, endothelial cell; Ch, vascular channel; L, lymphocyte; S, supporting cells.

Under the transmission microscope, the endothelial cells lining the sheathed capillary were plump and cuboidal in appearance (Figure 2a). The vascular channel presented between the adjacent endothelial cells (Figure 2b). Approaching the discontinuous basement membrane, the vascular channel, which extended to the ellipsoid, formed (Figure 2c). The supporting cells arranged around the endothelial cells were pale in color. The lymphocytes appeared outside of the supporting cells along the vascular channel from the discontinuous basement membrane to the ellipsoid (Figure 2c).

### VCAM-1 and MADCAM-1 expression in the chicken spleen

**Figure 3:**
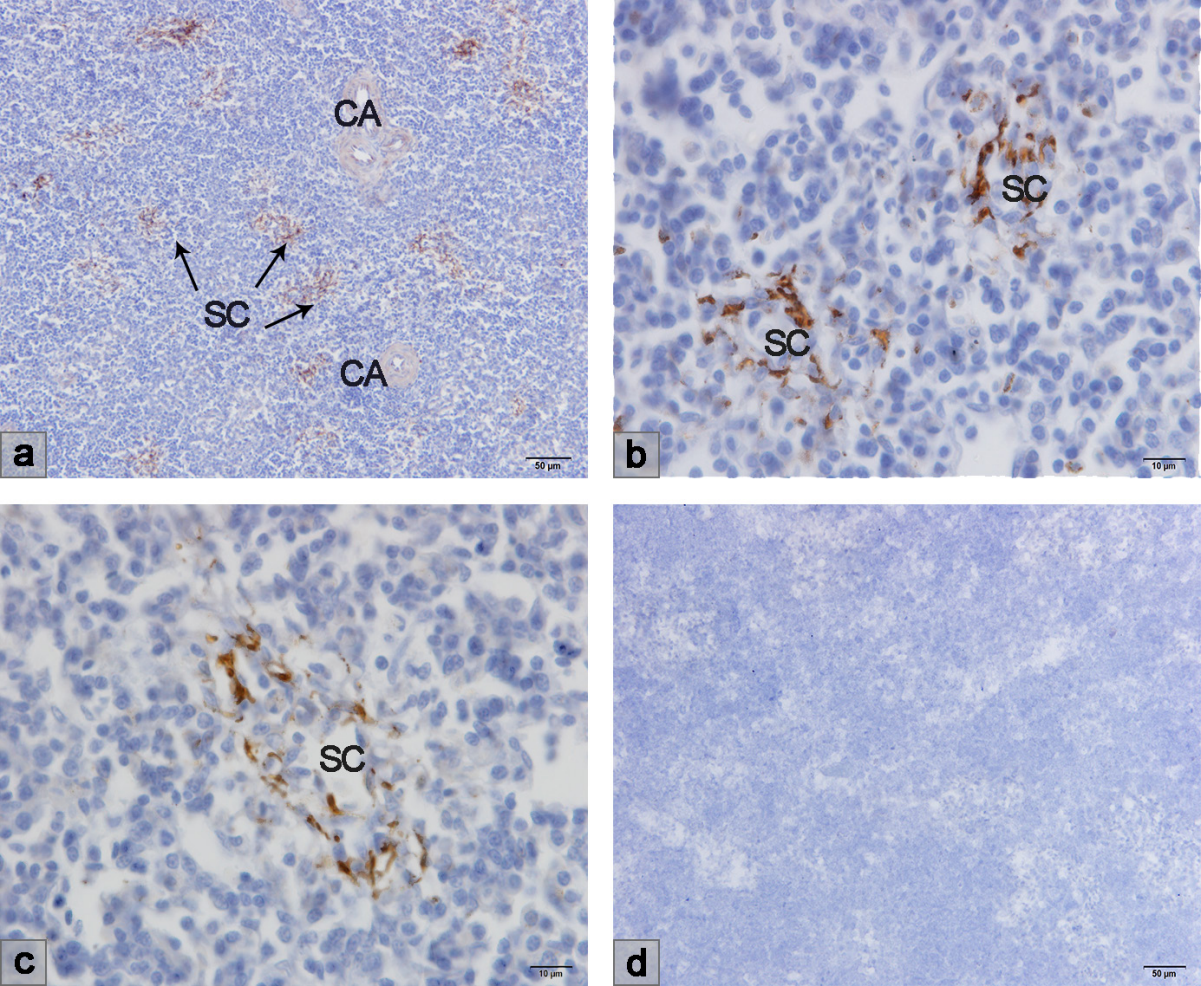
Immunohistochemical result of VCAM-1 mAb **a**.-**c**. VCAM-1 was expressed on the sheathed capillary but was negative on the central artery. **d**. Negative control. SC, sheathed capillary; CA central artery.

**Figure 4:**
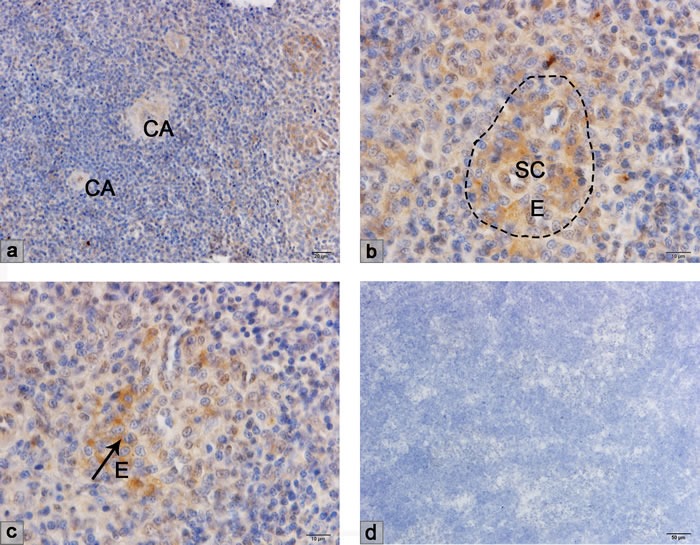
Immunohistochemical result of MADCAM-1 mAb **a**.-**c**. MADCAM-1 was expressed on the ellipsoid and the sheathed capillary but was negative on the central artery. CA, central artery; E, ellipsoid.

VCAM-1 and MADCAM-1 expressed on the endothelium are the ligands of adhesion molecules VLA-4 (α_4_β_1_) and α_4_β_7_ expressed on lymphocytes. The immunohistochemical results showed VCAM-1 expression on the sheathed capillary, but were negative on the central artery (Figure 3). MADCAM-1 was expressed on the site of the ellipsoid as well as the sheathed capillary, but not on the central artery (Figure 4).

### CFSE-labeled lymphocytes homing to the chicken spleen

**Figure 5:**
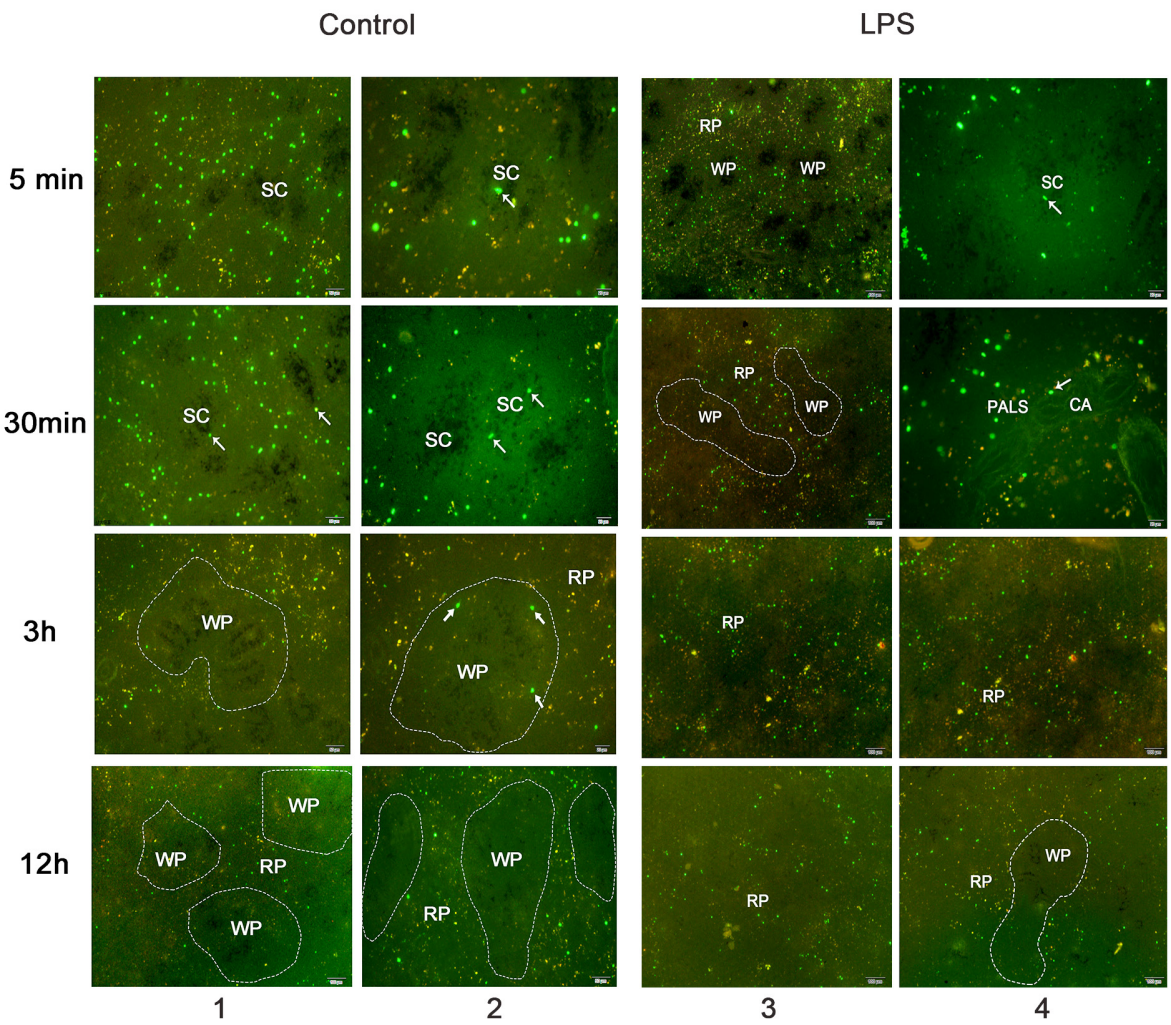
CFSE labeled lymphocytes transferred to the chicken spleen in the control and LPS group After 5 min, 30 min, 3 h, and 12 h, the labeled lymphocytes (arrow) distributed in the sheathed capillary, in the white pulp and the red pulp. The outlined area indicated the white pulp of the chicken spleen. The black area with the carbon injection indicates the splenic ellipsoid. 1 and 3 panel indicated the low magnification of the CFSE labeled lymphocyte after transferring. 2 and 4 panel indicated the higher magnification of the CFSE labeled lymphocyte after transferring. SC, sheathed capillary; WP, white pulp; RP, red pulp; CA, central artery; PALS, periarteriolar lymphatic sheath.

In our study, the host chickens were sacrificed 5 min, 30 min, 3 h and 12 h after the lymphocyte transfer. CFSE (5, 6- carboxyfluorescein diacetate, succinimidyl ester) labeled lymphocytes were distributed in the host chicken spleen (Figure 5). Carbon injection was used to determine the area of the sheathed capillary and the ellipsoid. Five minutes after transferring, some of the labeled lymphocytes were observed inside the sheathed capillary, and some appeared outside of the carbon area ellipsoid. Others were present in the PELS of the white pulp and the red pulp. Thirty min after the transfer, the CFSE labeled lymphocytes were present in the ellipsoid, and most were in the PELS and the red pulp. Three hours after the transfer, the CFSE labeled lymphocytes were mostly present in the PELS and the red pulp. However, until twelve hours after the transfer, most were present in the red pulp area, fewer appeared in the white pulp, and the number of labeled lymphocytes in the spleen was significantly reduced.

Compared with lymphocytes homing to the normal spleen, the CFSE labeled lymphocytes also migrated to the LPS treated spleen through the sheathed capillary. Five minutes after the transfer, lymphocytes were observed in the sheathed capillary, the white and red pulp. After thirty minutes, the labeled lymphocytes were mostly distributed in the red pulp, the surrounding of central artery PALS, and there were few lymphocytes in the white pulp, especially in the ellipsoid area. After 3 h and 12 h, the CFSE labeled lymphocytes distributed irregularly in the red pulp, and the number of lymphocytes presented in the spleen was significantly decreased.

### LPS-induced lymphocyte migration in the chicken spleen

**Figure 6:**
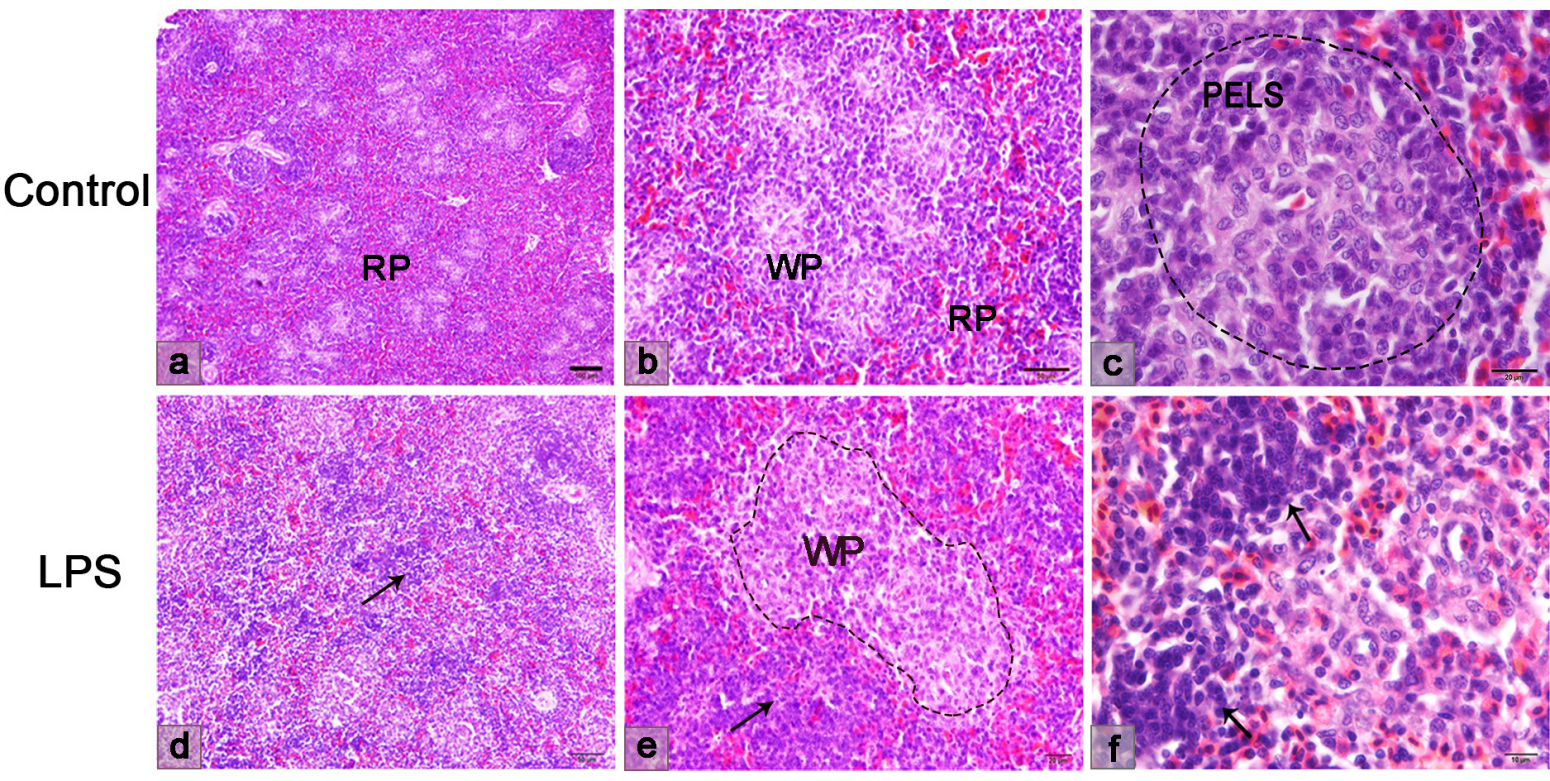
Histological structure of the chicken spleen **a**.-**c**. The normal spleen structure. The red and white pulps are clearly distinguished. **d**.-**f**. The spleen structure after LPS injection. Lymphocytes irregularly presented in the red pulp (arrow), but disappeared in the PELS of white pulp. WP, white pulp; RP, red pulp; PELS, periellipsoidal lymphatic sheath.

By HE staining, LPS injection affected lymphocyte migration in the chicken spleen (Figure 6). Three hours after an intraperitoneal injection of LPS, the area of the white pulp changed. Lymphocytes were absent from the PELS of the white pulp under inflammation. The ellipsoids were exposed in the red pulp without the surrounding PELS. The lymphocytes in the PELS mostly migrated to the red pulp compared to the normal spleen.

### LPS induced lymphocyte homing to the chicken spleen through the cuboidal endothelial cells of the sheathed capillary

**Figure 7:**
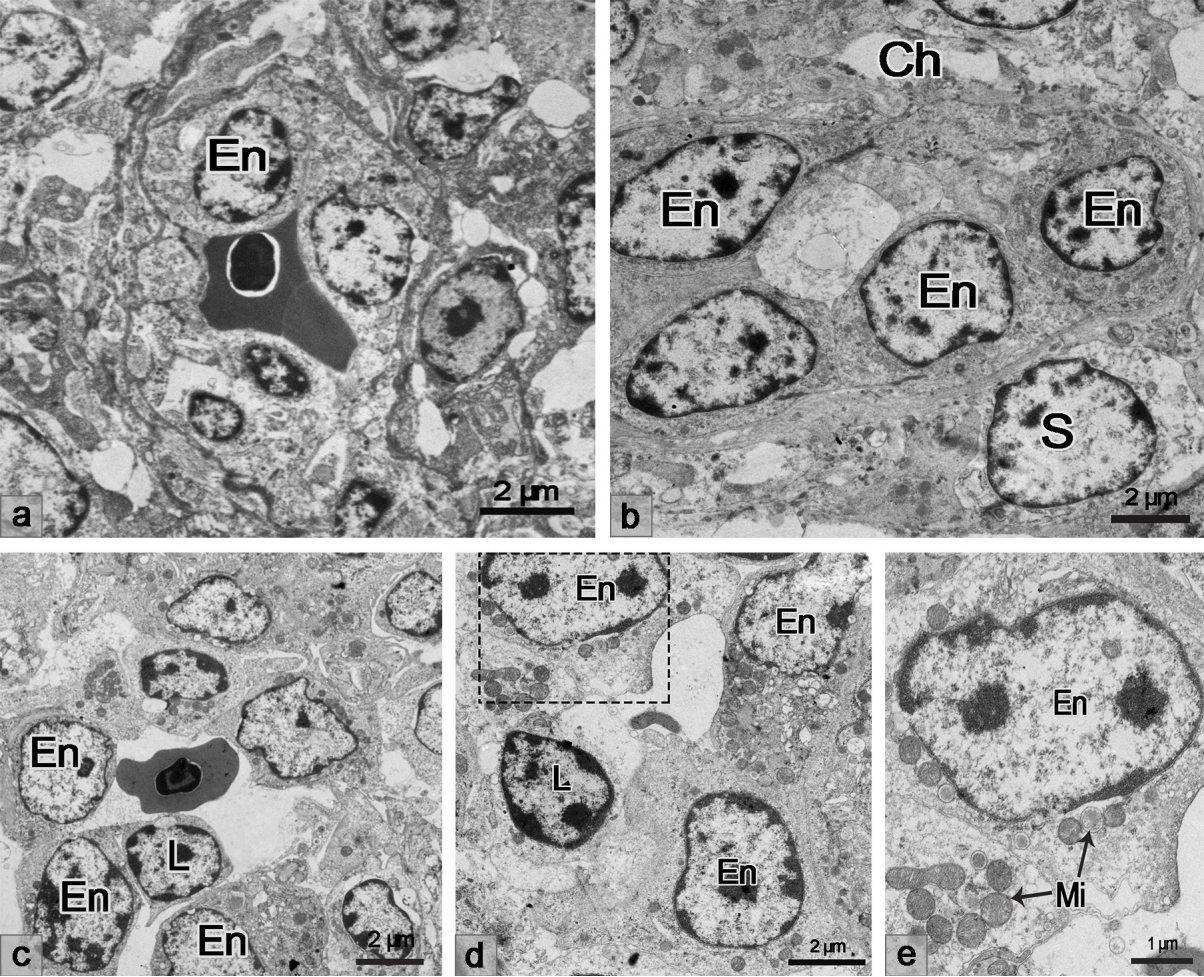
TEM showing the endothelial cells of the sheathed capillary **a**. Endothelial cells of the sheathed capillary were cuboidal in appearance. **b**. The vascular channel was present outside of the endothelial cells of the sheathed capillary. **c**. Lymphocytes adhered to the endothelial luminal surface. **d**. Lymphocytes traversed to the basal surface of the capillary. **e**. Higher magnification of the rectangular area. The cytoplasm of the endothelial cells contained abundant mitochondria. En, endothelial cell; Ch, vascular channel; L, lymphocyte; S, supporting cells; Mi, mitochondria.

Ultrastructurally, the endothelial cells of the sheathed capillary also showed a cuboidal appearance after the LPS treatment (Figure 7). The endothelial cells exhibited relatively loose cellular connections (Figure 7a and 7c). The structure of the spleen ellipsoid was not compact when there was lymphocyte transendothelial migration. The vascular channel was present outside of the endothelial cells of the sheathed capillary (Figure 7b). In our observation, lymphocytes in the blood circulation adhered to the endothelial luminal surface and then passed through the basal surface of the capillary (Figure 7c and 7d). The cytoplasm of the endothelial cells contained abundant mitochondria when the lymphocytes migrated through the adjacent endothelial cells (Figure 7e).

### LPS-induced CD3+ T and Bu-1+ B cells redistribution in the chicken spleen

**Figure 8:**
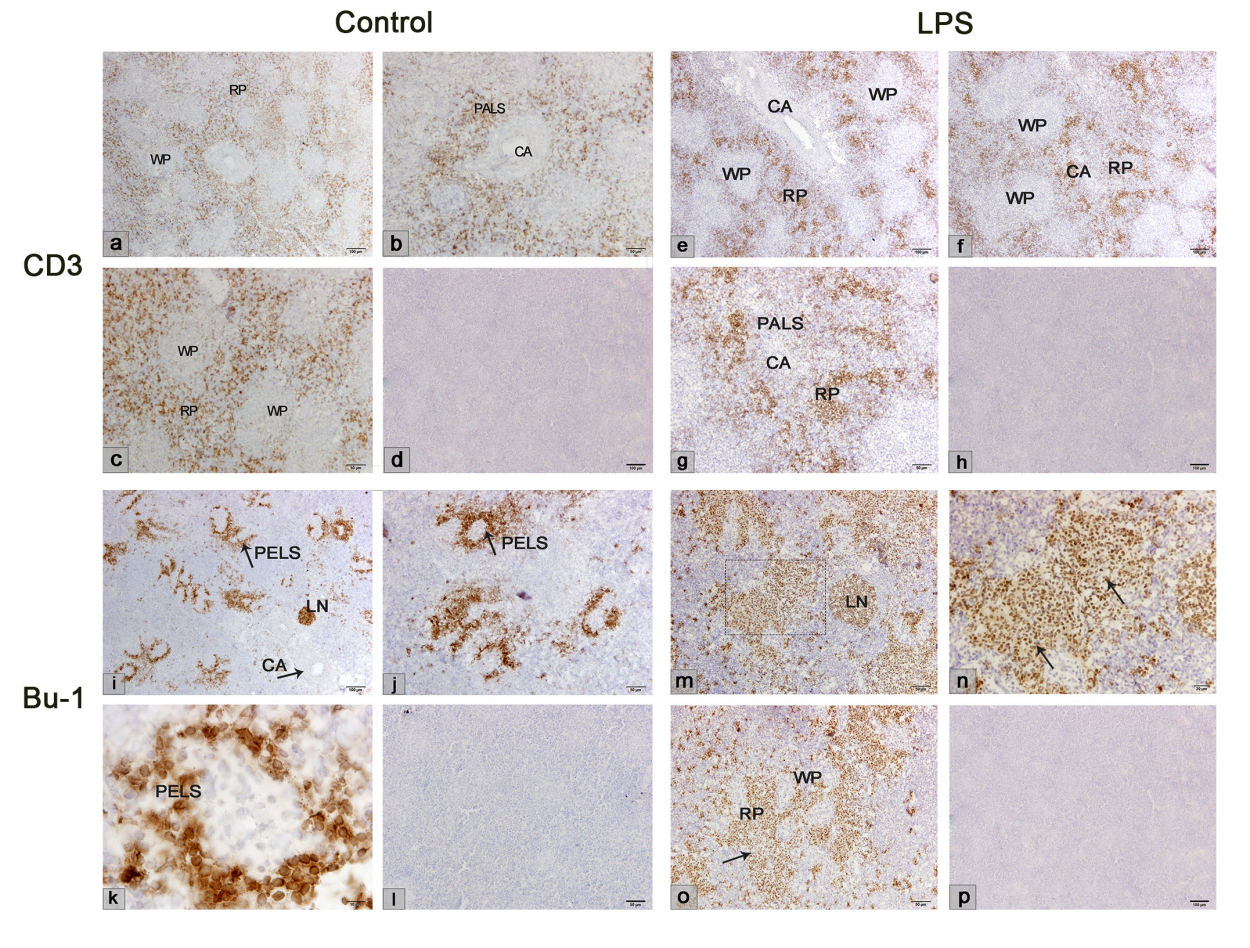
Immunohistochemical results of chicken CD3 and Bu-1mAb in the control and LPS chicken spleen **a**.-**c**. CD3+ cells expressed on the red pulp in the control spleen. **e**.-**g**. CD3+ cells were located in the red pulp and fewer were in the PALS in the LPS chicken spleen. **i**.-**k**. Bu-1+ cells expressed on the PELS and the LN in the control chicken spleen. **m**.-**o**. Bu-1+ cells accumulated in the red pulp (outlined rectangle) and the lymph nodules. **d**., **h**., **l**., **p**. Negative control. WP, white pulp; RP, red pulp; CA, central artery; LN, lymph nodule; PALS, periarteriolar lymphatic sheath; PELS, periellipsoidal lymphatic sheath.

**Figure 9:**
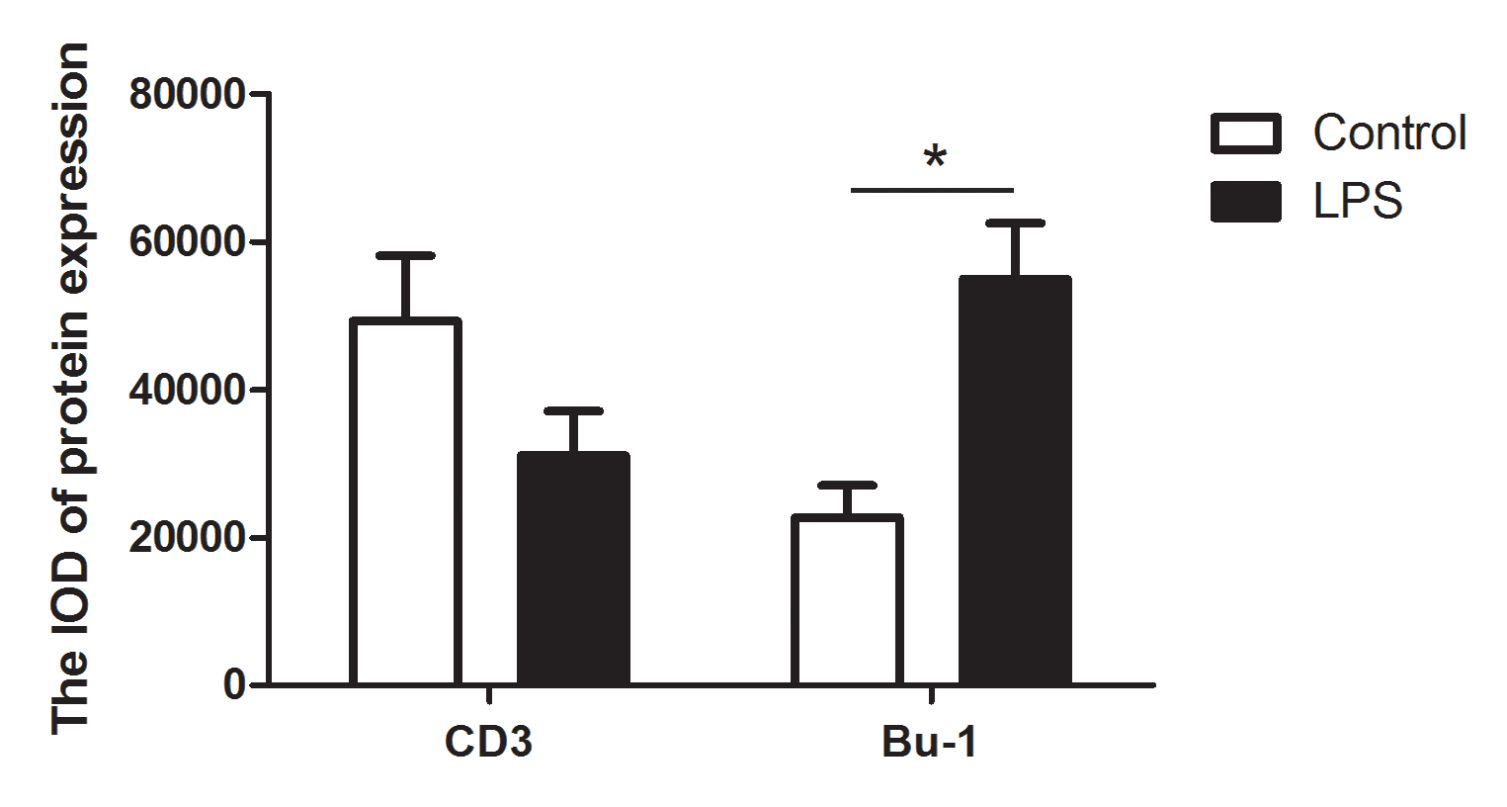
The integral optical density (IOD) of the CD3+ and Bu-1+ cells in the control and LPS chicken spleen The values are the mean ± SEM. *P < 0.05, the LPS group compared with the control group.

The distribution of T and B lymphocytes in the chicken spleen was determined by immunohistochemical staining. In the chicken, CD3 and Bu-1 mAb are the major markers of the T and B cell surfaces, respectively. In the normal chicken spleen, the CD3+T cells were located in the PALS and the red pulp (Figure 8). After LPS stimulation, the CD3+ T cells mostly distributed in the red pulp and fewer distributed in the PALS. The Bu-1+B cells were mostly located in the PELS, and fewer distributed in the red pulp in the normal chicken spleen; however, after LPS stimulation, the Bu-1+ B cells appeared to migrate to the red pulp and the lymph nodule but were absent from the PELS. The integral optical density (IOD) of CD3+ and Bu-1+ revealed that the number of CD3+ T cells decreased in the LPS-stimulated spleen, while the number of Bu-1+ B cells significantly increased after the LPS injection (Figure 9).

### LPS increased the expression of IL-6 and TNF-α

**Figure 10:**
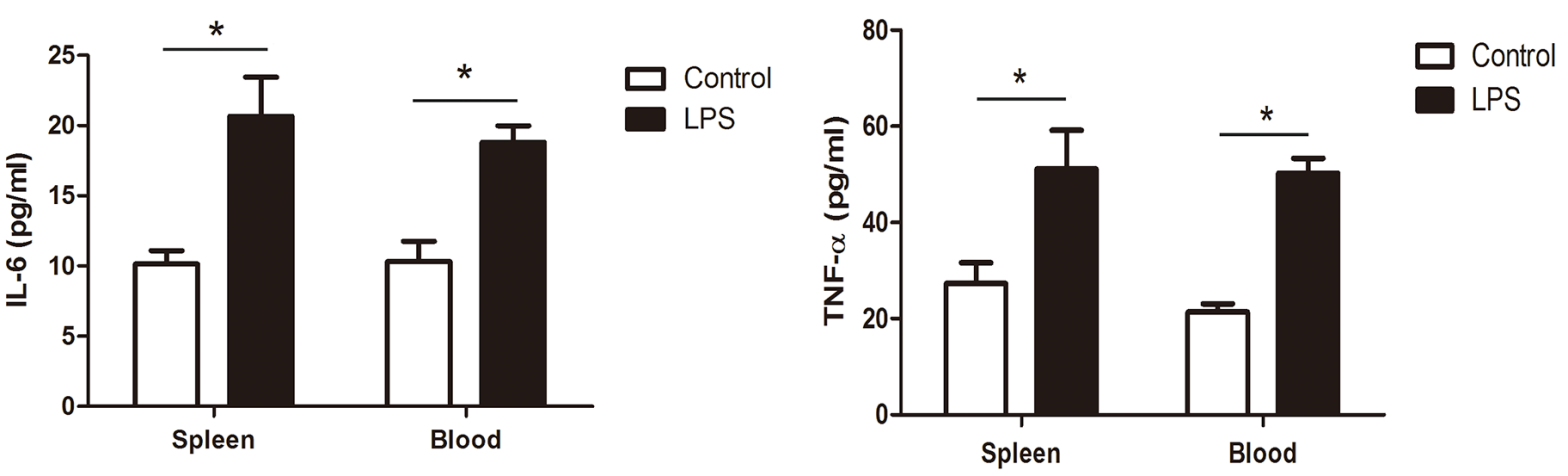
The concentrations of IL-6 and TNF-α in the chicken spleen and blood by an ELISA assay The values are the mean ± SEM. *P < 0.05, the LPS group compared with the control group.

The LPS-induced inflammatory immune response is mediated through the Toll-like receptor pathway, resulting in the increased expression of cytokines, such as IL-6 and TNF-α. In our study, the concentrations of IL-6 and TNF-α were determined in the chicken spleen and blood by an ELISA assay. Three hours after the LPS injection, the expression of IL-6 and TNF-α in the spleen increased compared with the normal spleen. Meanwhile, the expression of IL-6 and TNF-α in the blood was also up-regulated in the LPS-treated chicken (Figure 10).

### LPS increased integrin β1, VCAM-1, ILK and AKT mRNA expression

**Figure 11:**
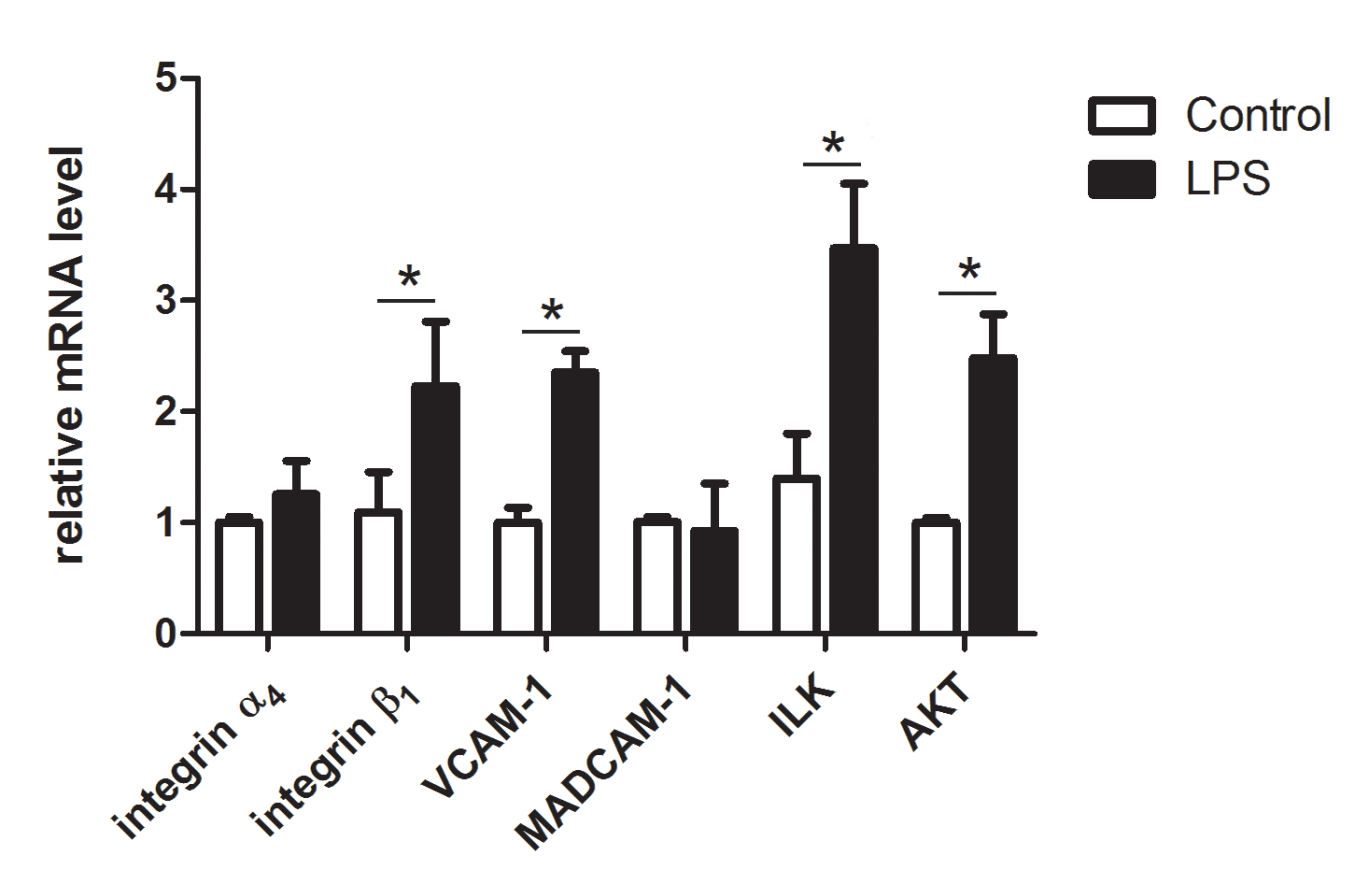
qPCR of the mRNA expression of the lymphocyte homing related genes in chicken spleen after LPS treatment Integrin β_1_, VCAM-1, ILK and AKT were significantly increased with the LPS treatment (*P* < 0.05), while integrin α_4_ and MADCAM-1 were not affected. The values are the mean ± SEM. *P < 0.05, the LPS group compared with the control group.

To investigate whether LPS regulates lymphocyte homing to the chicken spleen, the mRNA expressions of the adhesion molecules integrin α_4_, integrin β_1_, VCAM-1, and MADCAM-1 were examined by qPCR (Figure 11). LPS challenge up-regulated integrin β_1_ and VCAM-1 but did not affect integrin α_4_ and MADCAM-1 mRNA expression. VCAM-1 is the specific lymphocyte homing addressin of integrin β_1_. To further study the effect of integrin β_1_-VCAM-1, the integrin-linked kinase (ILK) and the PI3K/AKT signaling pathway were investigated. In our study, the mRNA of ILK and AKT increased after LPS treatment.

### LPS increased integrin β1, VCAM-1, ILK and AKT pSer473 protein expression

**Figure 12:**
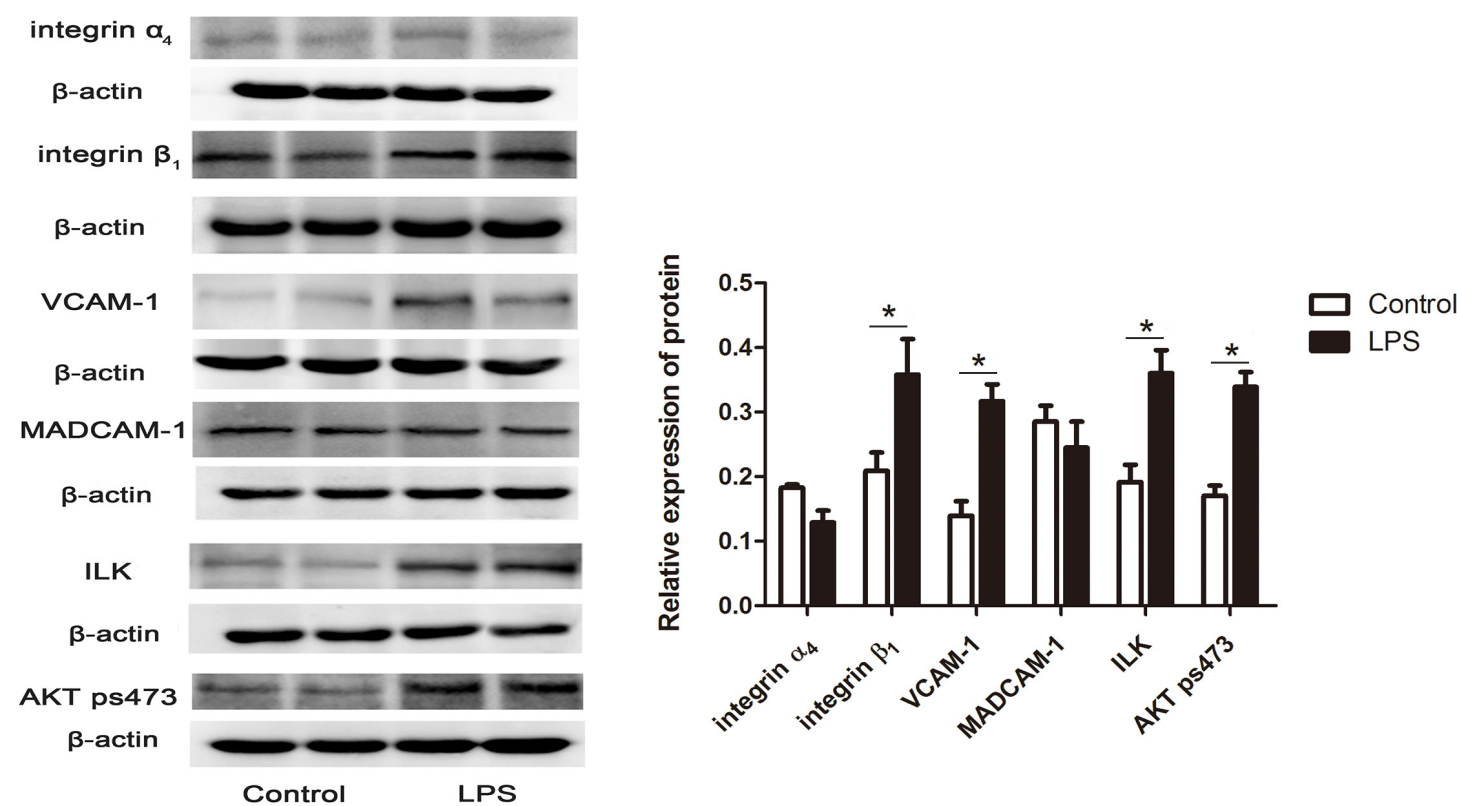
Western blotting for LPS-induced integrin α 4, MADCAM-1, integrin β1, VCAM-1, ILK and AKT pSer473 protein expression. The histogram represents the densitometric analysis of the Western blotting. The values are the mean ± SEM. *P < 0.05, the LPS group compared with the control group.

Western blotting was used to detect the immunoreactivity of integrin α_4_, integrin β_1_, VCAM-1, MADCAM-1, ILK and AKT pSer473 in the chicken spleen after LPS treatment. β-actin was used as an internal control in the experiment (Figure 12). In our study, integrin α_4_ was detected as an immunoreactive band at approximately 115 kDa, which was consistent with its protein expression. Integrin β_1_ was observed at 88 kDa, VCAM-1 was observed at 81 kDa. MADCAM-1 was observed at 43 kDa, and ILK and phosphorylated AKT Ser473 (AKT pSer473) were observed at approximately 59 kDa and 60 kDa, respectively. β-actin was observed at approximately 40 kDa. From these positive expressions, the proteins corresponding to integrin β1, VCAM-1, ILK and AKT pSer473 were significantly increased after LPS injection (*P* < 0.05); however, there was no significant variation in the protein expressions of integrin α_4_ and MADCAM-1 between the LPS treatment and the control group, which was consistent with the results of mRNA expressions. Compared with the expression of MADCAM-1, integrin α_4_ showed a weak positive expression in the chicken spleen.

## DISCUSSION

The avian immune system and the immune response show significant differences compared with the mammalian immunology. As the largest peripheral lymphoid organ, chicken spleen plays a crucial role in immune responses, particularly to blood-borne antigens [2, 3]. In the present study, we investigated the cytological characterization of the chicken spleen. The endothelial cells of the sheathed capillary of the chicken spleen were cuboidal in appearance, which was consistent with a previous study [14]. Lymphocytes were found in the splenic ellipsoid approaching the vascular channel of the sheathed capillary. The vascular channel and the loose cellular connection between the adjacent endothelial cells probably facilitate the passage of the lymphocyte migration from the bloodstream to the splenic white pulp in the chicken spleen. Moreover, the tissue-specific vascular addressins on the endothelium, VCAM-1 and MADCAM-1, were expressed on the sheathed capillary and the ellipsoid, which further confirmed the HEV-like vessels of the sheathed capillary.

Furthermore, the adoptive cell transfer revealed lymphocyte homing to the chicken spleen in this study. The CFSE labeled lymphocytes containging T and B lymphocytes were distributed in the branching sheathed capillary, in the ellipsoid and were present in the PELS, PALS and red pulp. With the time course of migration into the chicken spleen, most of lymphocytes were present in the red pulp. Few lymphocytes were found in the ellipsoid. CFSE labeled lymphocytes transferring demonstrated lymphocytes homing and their distribution. Immunohistochemical staining of CD3 and Bu-1 in the chicken spleen suggested that T and B lymphocytes distributed in different compartments of the chicken spleen. The CD3+T cells were located in the PALS and the red pulp, and the Bu-1+B cells were mostly located in the PELS surrounding the ellipsoid. From the adoptive cell transfer and immunohistochemical results, we can speculate that B lymphocytes transit to the spleen in the direction from the blood to the white pulp ellipsoid, and a few pass into the red pulp. T lymphocytes mostly migrate from the ellipsoid to the PALS and then pass into the red pulp, which is different from the circulation in the mammalian spleen. Mitchell reported that lymphocytes in the white pulp enter the arterioles and circulate to the red pulp *via* the marginal zone in the mammalian spleen [32, 33]. Without the marginal zone, the sheathed capillary embedded in the chicken ellipsoid is the HEV-like vessels, which provides the structural basis for lymphocyte migration from the bloodstream to the white and the red pulp. The decreased lymphocytes ultimately present in the red pulp are destined to enter the venous circulation [34, 35].

To further investigate lymphocyte homing in the chicken spleen during LPS stimulation. LPS-induced inflammation was successfully performed in our study. The inflammatory cytokines IL-6 and TNF-α were examined, and increased expression was observed in the chicken spleen and peripheral blood. It has been reported that cytokines TNF-α induced MADCAM-1 increasing and increases adhesiveness of endothelium for lymphocytes [36, 37]. Whether LPS simulation induced lymphocyte homing and its adhesion molecules in the sheathed capillary of chicken spleen? From our results, we found that LPS activated the endothelium of the sheathed capillary. Electron microscopy showed that the lymphocytes migrated to the chicken spleen through the cuboidal endothelial cells of the sheathed capillary under LPS treatment. The weak endothelial cell junctions and the activated endothelial cells provided the structural basis for lymphocyte migration. Two pathways of lymphocyte transmigration of the endothelium, transendothelial and intraendothelial migration, are reported [38]. Our findings suggested that the lymphocytes transmigrated the adjacent endothelial cells of the sheathed capillary to the inflammation-induced chicken spleen. The results of the CFSE lymphocyte transfer verified that the lymphocytes were homing to the LPS-treated chicken spleen from the sheathed capillary to the ellipsoid and the red pulp. The HE staining and the immunohistochemical results showed that B and T lymphocytes redistributed in the chicken spleen after LPS injection. The B lymphocytes tend to migrate from the PELS to the red pulp, while the T lymphocytes decreased in the red pulp, which might be a result from the activation of the humoral immunity after the LPS stimulation.

The molecular mechanism of lymphocyte homing to the mammalian lymph nodes has been well studied and involves several families of molecules, such as chemokines and integrins [39–42]. There is limited evidence reporting that integrin supports the firm adhesion between the lymphocyte and associated endothelium in the murine spleen [43]. Lo et al found the integrins LFA-1 and VLA-4 function to promote lymphocyte transit from the marginal zone into the white pulp cords in the murine spleen [44]. But such information regarding the molecular mechanism underlying lymphocyte homing to the chicken spleen is lacking. In our study, LPS treatment activated integrin β_1_ and VCAM-1 in the chicken spleen at the mRNA and protein levels and also resulted in enhanced integrin linked kinase (ILK) and phosphorylated AKT Ser473 expression. The morphological characteristics and the molecular expression suggest that LPS activates the endothelium and recruits lymphocytes to migrate to the spleen red pulp.

ILK is an serine-threonine protein kinase capable of interacting with the cytoplasmic domains of integrin β1, β2, and β3 subunits. ILK is involved in the expression of cell adhesion molecules by endothelial cells activated with the inflammatory stimulus LPS [45–47], and it is an upstream effector of the Pi(3)K-dependent regulation of protein kinase B (PKB/AKT), which phosphorylates PKB/AKT on serine-473 to promote the cell cycle and migration [48–50]. From our results, LPS activates the endothelium by up-regulating the adhesion molecule VCAM-1, B Lymphocytes migrate and accumulate in the red pulp, and ILK phosphorylates PKB/AKT on serine-473 to promote cell migration to the inflamed compartment. This is the first study to demonstrate lymphocyte homing in the chicken spleen after LPS-induced inflammation. In the past ten years, Nolte et al [51] and Lo et al [44] investigated the regulation of lymphocyte migration to the splenic white pulp in rodents, and there may be some molecular mechanism differences of lymphocyte homing to the spleen between the chicken and rodent. The detailed mechanisms underlying T and B lymphocyte homing to the chicken spleen need to be further explored. Further studies of the mechanism controlling lymphocytes entering into the lymphoid compartment of the chicken spleen will contribute to a better understanding of avian immunology and provide new targets for immunomodulatory drug therapies in infectious disease.

## MATERIALS AND METHODS

### Animals

Adult male and female Sanhuang broiler chickens, 60 days of age and weighing 1.0-1.5 kg, were used for this study. Immunohistochemistry, transmission electron microscopy, adoptive cell transfer, quantitative real-time PCR and Western blot were undertaken in each group of chickens (*n* = 5 per group).

The chickens were randomly divided into the control (0.01 M phosphate-buffered saline, PBS, pH 7.4) and the LPS groups. The chickens in the LPS group were injected intraperitoneally with LPS from Escherichia coli 055:B5 (L2880, Sigma, St Louis, MO, USA ) at 2 mg/kg body weight. The chickens in the control group were injected with the same dose of PBS. Three hours after injection, the spleen was collected immediately for further experiments.

All of the animals were euthanized by cervical dislocation after intravenous administration of 3% sodium pentobarbital (25 mg/kg). All of the procedures were approved by the Nanjing Agricultural Veterinary College Experimental Animal Ethics Committee (Approval number SYXK (SU) 2010-0005; Date of approval, 18 June 2010).

### Light microscopy

The spleen samples for the control and LPS groups were fixed in 4% buffered paraformaldehyde and embedded in paraffin wax. Serial sections (at 5 μm) were obtained and were stained with hematoxylin and eosin (HE) for light microscopy using an Olympus microscope (BX53) with a camera (Olympus DP73).

### Transmission electron microscopy (TEM)

The normal spleen samples were obtained immediately post-mortem. Small samples were cut into 1 mm^3^ blocks, immersed in 2.5% glutaraldehyde fixative in 0.01 M phosphate-buffered saline (PBS; pH 7.4) at 4 °C overnight and were then submerged in 1% osmium tetroxide in the same buffer for 60 min. The samples were dehydrated in ascending concentrations of ethyl alcohol, infiltrated with a propylene oxide-Araldite mixture, and embedded in Araldite. Ultrathin sections (50 nm) were stained with uranyl acetate and lead citrate for 20 min each. The sections were examined and photographed with a transmission electron microscope H-7650 (Hitachi).

### Immunohistochemistry

The spleen samples from the control and LPS groups were fixed in 4% buffered paraformaldehyde for over 24 h and were embedded in paraffin. The samples were sectioned at a 5 μm thickness on poly-L-lysine coated slides and were stained according to the immunohistochemical methods. Briefly, after deparaffinization and washing in phosphate-buffered saline (PBS), the sections were inactivated with endogenous peroxidase by covering them with 3% hydrogen peroxide for 10 min. Then, the sections were blocked with 5% bovine serum albumin and were incubated with the primary anti-chicken antibodies CD3 and Bu-1 at a 1:150 dilution (Southern Biotech, USA) at 4 °C overnight. After washing in PBS, the sections were incubated with a biotinylated goat anti-rabbit IgG (Boster Biotechnology) for 1 h at 37 °C. The sections were then washed in PBS and incubated with an avidin-biotinylated peroxidase complex for 30 min at 37 °C. After washing in PBS, the activity was revealed with DAB (Boster Biotechnology). After PBS washing, the sections were counterstained with hematoxylin. The negative control with PBS was used instead of the primary antibodies.

The cell count measurements of the immunohistochemical staining results were performed using Image-Pro Plus 6.0 (Media Cybernetics, Silver Spring, MD). The positive areas are expressed as the integral optical density (IOD). The mean result of six areas per slide was used to calculate the IOD.

### Adoptive cell transfer experiment

The lymphocytes isolated from the spleen cell suspension from 60 day chickens in 0.01 M phosphate-buffered saline (PBS; pH 7.4) were incubated for 37 °C for 10 min at 2× 10^7^ cells/ml with CFSE at a final concentration of 5 μM. The lymphocytes (2×10^7^ cells) labeled with CFSE were then injected into the host chickens (normal and LPS chickens) through the heart. The host chickens were sacrificed 5 min, 30 min, 3 h and 12 h after the lymphocyte transfer. Each time group was performed in three biological replicates. A carbon injection was performed 20 min before sacrificing the chickens. Blocks of the fresh spleen tissue were collected and stored in liquid nitrogen. Cryostat sections were prepared using a Jung-2700 freezing microtome. The sections were washed in PBS for 5 min three times and were then air-dried for fluorescence microscopy observations using an Olympus microscope (BX53) with a camera (Olympus DP73).

### IL-6 and TNF-α assay

IL-6 and TNF-α were measured from the blood and spleen homogenate supernatant by using a commercial ELISA kit (R&D Systems, USA). The blood serum and spleen homogenate supernatant were diluted and studied using a standard curve. The diluted samples were paralleled to the standard curve to indicate the validity of the assay. The ELISA procedure was performed according to the manufacturer's instructions. All of the samples were measured in duplicate.

### Primer design

The primer sequences (Table S1 in supplemental data) were designed by the method of the sequences identified through the bioinformatics approach and referred to the NCBI database. Primer Premier 3.0 software was used for the primer design using the following parameters: a primer length of 20 to 24 nucleotides; primer melting temperature of 58-60 °C; the guanine and cytosine content of the primers was 40% to 60%; the difference in the melting temperature between the forward and reverse primers was 1 °C to 2 °C; and the amplification length was 100-200 base pairs (bp). All of the primers under the parameters were designed to facilitate amplification and BLAST searches against chicken DNA sequences in GenBank to ensure amplification specificity.

### RNA isolation and qPCR analysis

RNA was extracted from the chicken spleen using the Trizol reagent (Invitrogen, Carlsbad, CA, USA) according to the manufacturer's instructions. The RNA concentrations were determined using BioPhotometer Plus (Eppendorf, German) with a spectrophotometric analysis. The RNA samples with an OD260/280 ratio between 1.8 and 2.0 were used for further examination. The RNA were reverse transcribed into cDNA according to the SuperScript First-Strand Synthesis System (Invitrogen, Carlsbad, CA, USA). Quantitative real-time PCR (qPCR) was performed using the MyiQ2 Real-Time PCR System (Bio-Rad, California, USA). The PCR system was performed in a 20 μL volume containing SYBR Green qPCR Supermix (Invitrogen, Carlsbad, CA, USA), 10 mM specific primer and 0.1 mg of the template cDNA. Each sample was performed in three biological replicates. The reactions were carried out with an initial denaturation step of 95 °C for 10 s followed by 40 cycles of 95 °C for 5 s, 60 °C for 30 s and 72 °C for 10 s. The relative expression of the target genes was referred to β-actin and was analyzed using the 2^-^△△^Ct^ method [52].

### Western blot analysis

The mAbs of integrin α_4_ (BS6061, Bioworld Technology Inc), integrin β_1_ (orb10944, Biorbyt, UK), VCAM-1(11444-1-AP, Proteintech, China), MADCAM-1 (21917-1-AP, Proteintech, China), ILK (BS6638, Bioworld Technology Inc), AKT pSer473 (BS4007, Bioworld Technology Inc) and β-actin (AP0060 Bioworld Technology Inc) were studied as the primary antibodies in the Western blotting. Proteins were extracted from chicken spleen with cold lysis buffer (50 mM Tris/HCl (pH 7.6), 150 mM NaCl, 1% Nonidet-P40, 1% sodium deoxycholate, 0.1% SDS and 0.05 mM PMSF) for 30 min. The protein concentrations were determined with the BCA Protein Assay kit (Thermo Fisher Scientific, USA). The same amounts of protein (30 μg/lane) were subjected to an 8% SDS-PAGE gel, and the separated proteins were transferred onto polyvinylidene di-fluoride (PVDF) (Millipore, Bedford, MA) membranes. The membranes were blocked with 5% non-fat milk and were incubated at 4 °C overnight with the primary antibody. Peroxidase-labeled anti-rabbit IgG (1:5000, BS13278, Bioworld Technology Inc., Louis Park, MN) was incubated as the secondary antibody for 1-2 h. The positive signal was detected by an enhanced chemiluminescence detection system (Vazyme Biotech, China). The data of the protein expression differences were analyzed by Quantity One software (Bio-Rad, USA). The content of the target proteins is described as the fold change relative to the average content of the control group.

### Statistical analysis

The data were analyzed with GraphPad Prism 5 software (San Diego, CA) and expressed as the means ± SEM. The data analysis was performed using SPSS software version 16.0 with a Duncan's test and a one-way analysis of variance (ANOVA). The differences were considered statistically significant at *P* < 0.05.

## SUPPLEMENTARY MATERIALS TABLES


