# Supplementary material for: Cytological study on the regulation of lymphocyte homing in the chicken spleen during LPS stimulation
Source: Oncotarget. 2017 Jan 4;8(5):7405–19. doi: 10.18632/oncotarget.14502 (PMC5352331; doi:10.18632/oncotarget.14502)
Supplement: Supplementary file 1 [file oncotarget-08-7405-s001.pdf]

Cytological study on the regulation of lymphocyte homing in the chicken spleen during LPS stimulation

Qian Zhang[1,2], Yasir Waqas[1], Ping Yang[1], Xuejing Sun[1], Yi Liu[1], Nisar Ahmed[1], Bing Chen[1], Quanfu Li[1], Lisi Hu[1], Yufei Huang[1], Hong Chen[1], Bing Hu[3] and Qiusheng Chen[1]

[1] Laboratory of Animal Cell Biology and Embryology, College of Veterinary Medicine, Nanjing Agricultural University, Nanjing, China

[2] Key Laboratory of Antibody Techniques of Ministry of Health, Nanjing Medical University, Nanjing, China

[3] Biological experiment and Teaching Center, College of Life Sciences, Nanjing Agricultural University, Nanjing, China

***Correspondence to**: Qiusheng Chen, **email**: chenqsh305@njau.edu.cn*

Keywords: lymphocyte homing; chicken spleen; HEV; sheathed capillary; adhesion molecules; Immunology and Microbiology Section, Immune response, Immunity

Received: *May 22, 2016* ***Accepted***: *December 27, 2016* ***Published***: *January 04, 2017*

Supplementary Table 1: Primer sequences used in the qPCR analysis

Gene	GenBank accession number	Forward Primer<br>Reverse Primer	Amplicon length (bp)
Integrin α4	XM421974	CAGAGAATACCAGCACAG<br>CACAGCAGAATCGGATAG	132
Integrin β1	XM015281260	TCTCTGTTGCTTGATATGG<br>TGTTGGTTCTCCTTCTTG	151
VCAM-1	XM422310	TGTGAAGTAGTTAATCCTGTA<br>CTGAGCAAGAGATGGTAA	145
MADCAM-1	XM003642866	CACAGGGGCGGATCTTACAG<br>GTCTTGCTTGGAACGTGTGC	100
ILK	XM015280909	GGGACAAACTTCGTGGTGGA<br>CCTTGCAGTCATGTCCTCGT	153
AKT	NM205055	CCAAGAGACATTCCCGCCAT<br>CGGTCGTTCCTTGTAGCCAA	147
β-actin	NM205518	CCTGAACCTCTCATTGCCA<br>CCTGAACCTCTCATTGCCA	152
